# Pharmacokinetics of Novel Dopamine Transporter Inhibitor CE-123 and Modafinil with a Focus on Central Nervous System Distribution

**DOI:** 10.3390/ijms242316956

**Published:** 2023-11-29

**Authors:** Iva Spreitzer, Josefin Keife, Tobias Strasser, Predrag Kalaba, Jana Lubec, Winfried Neuhaus, Gert Lubec, Thierry Langer, Judith Wackerlig, Irena Loryan

**Affiliations:** 1 Department of Pharmaceutical Sciences, University of Vienna, 1090 Vienna, Austria; iva.spreitzer@univie.ac.at (I.S.); thierry.langer@univie.ac.at (T.L.); 2 Vienna Doctoral School of Pharmaceutical, Nutritional and Sport Sciences, University of Vienna, 1090 Vienna, Austria; 3 Translational Pharmacokinetics/Pharmacodynamics Group, Department of Pharmacy, Uppsala University, 75123 Uppsala, Sweden; 4 Programme for Proteomics, Paracelsus Medical University, 5020 Salzburg, Austriagert.lubec@lubeclab.com (G.L.); 5 Competence Unit Molecular Diagnostics, Center Health and Bioresources, AIT Austrian Institute of Technology GmbH, 1210 Vienna, Austria; winfried.neuhaus@ait.ac.at; 6 Department of Medicine, Faculty of Medicine and Dentistry, Danube Private University, 3500 Krems, Austria

**Keywords:** dopamine transporter inhibitor, *S*-CE-123, *R*-modafinil, neuropharmacokinetic, tissue distribution, metabolism

## Abstract

*S*-CE-123, a novel dopamine transporter inhibitor, has emerged as a potential candidate for cognitive enhancement. The objective of this study was to compare the tissue distribution profiles, with a specific focus on central nervous system distribution and metabolism, of *S*-CE-123 and *R*-modafinil. To address this objective, a precise liquid chromatography–high resolution mass spectrometry method was developed and partially validated. Neuropharmacokinetic parameters were assessed using the Combinatory Mapping Approach. Our findings reveal distinct differences between the two compounds. Notably, *S*-CE-123 demonstrates a significantly superior extent of transport across the blood–brain barrier (BBB), with an unbound brain-to-plasma concentration ratio (K_p,uu,brain_) of 0.5, compared to *R*-modafinil’s K_p,uu,brain_ of 0.1. A similar pattern was observed for the transport across the blood–spinal cord barrier. Concerning the drug transport across cellular membranes, we observed that *S*-CE-123 primarily localizes in the brain interstitial space, whereas *R*-modafinil distributes more evenly across both sides of the plasma membrane of the brain’s parenchymal cells (K_p,uu,cell_). Furthermore, our study highlights the substantial differences in hepatic metabolic stability, with *S*-CE-123 having a 9.3-fold faster metabolism compared to *R*-modafinil. In summary, the combination of improved BBB transport and higher affinity of *S*-CE-123 to dopamine transporters in comparison to *R*-modafinil makes *S*-CE-123 a promising candidate for further testing for the treatment of cognitive decline.

## 1. Introduction

Cognitive enhancement is one of the major aspects of neuropharmacology as cognitive decline is associated with aging and represents the main symptom for several neurodegenerative and psychiatric disorders, such as Alzheimer’s disease, Parkinson’s disease, schizophrenia, etc. [1,2,3,4]. According to recent studies and data, mild cognitive impairment is prevalent among adults aged 60 years or older in various regions [5,6,7]. Furthermore, the global prevalence of dementia is expected to almost double every 20 years, from 46.8 million in 2015 to 74.7 million in 2030 and 131.5 million in 2050, if similar trends persist [2,8]. The total estimated worldwide cost of dementia was US $818 billion in 2015 and is expected to rise to US $2.8 trillion by 2030 [8,9]. In this respect, neurotherapeutics for the treatment of cognitive decline are highly demanded.

Dopamine (DA) acts as a powerful regulator of different aspects of cognitive brain functions such as motor control, the modulation of affective and emotional states, reward mechanisms, reinforcement of behavior, and selected higher cognitive functions such as cognitive flexibility, executive functions, attention, etc. [10,11]. The dopamine transporter (DAT) plays an important role in the modulation of DA neurotransmission by driving the reuptake of extracellular DA into the presynaptic neurons [12]. This reuptake process terminates the signal and helps maintain the proper DA levels, preventing excessive or prolonged stimulation of receptors. Hence, normalizing DA transmission will contribute to improve cognitive decline not only related to neurologic or psychiatric diseases but also in normal aging [11].

**Figure 1:**
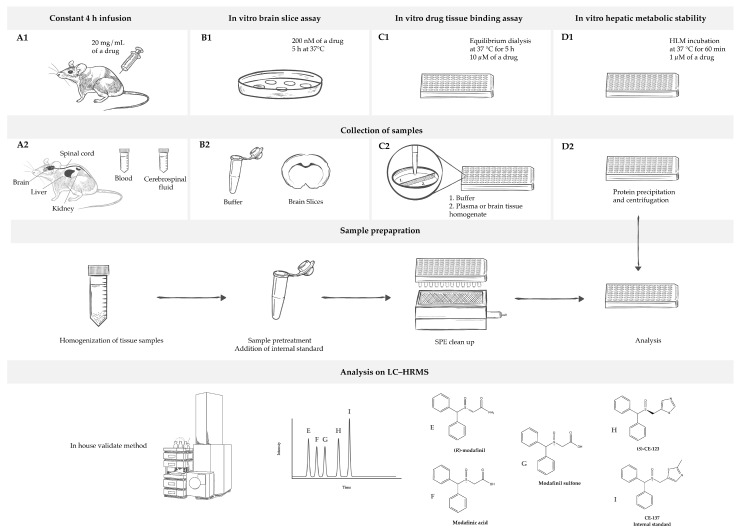
Overview of the study’s design. (**A1**) In vivo rat pharmacokinetic study measures of the total concentration of a drug in the blood, cerebrospinal fluid, and tissues (brain, spinal cord, liver, and kidneys) under steady-state conditions achieved using a 4 h continuous intravenous infusion of the drug. (**B1**) In vitro rat brain slice assay measures the total concentration of a drug in a brain slice and buffer under steady-state conditions established by incubating brain slices in the buffer with the drug. It is used for estimation of unbound volume of distribution in brain (V_u,brain_). (**C1**) In vitro drug tissue binding assay determines unbound fraction of the drug in plasma (f_u,plasma_) and brain homogenate (f_u,brain_). (**D1**) In vitro metabolic assay gives insight into stability of the drug in human liver microsomes (HLMs). For all sample collections ((**A2**–**C2**), with exception of (**D2**)), the preparation process includes tissue sample homogenization, sample pre-treatment, the addition of an internal standard (**I**), and a solid phase extraction (SPE) clean-up. However, for samples obtained in (**D2**), preparation involves protein precipitation followed by centrifugation. Afterward, samples are analyzed using liquid chromatography–high resolution mass spectrometry (LC–HRMS) by employing in-house developed and validated method. Analytes under analysis include *R*-modafinil (**E**), modafinic acid (**F**), modafinil sulfone (**G**), *S*-CE-123 (**H**), and CE-137 as an internal standard (**I**).

Given these implications, the DAT represents an attractive therapeutic target to enhance cognitive abilities. However, current DAT-targeting compounds, such as methylphenidate and several amphetamine-related drugs, have modest cognition-enhancing effects. Their use is also associated with addiction, impulsive behaviors, the potential induction of physiological changes that could lead to long-term transporter adaptations during addiction or therapeutic treatment regimens, etc. (for more reading, see [11,12,13,14,15]). In recent decades, an atypical DAT inhibitor, modafinil (2-[(Diphenylmethyl)sulfinyl]acetamide, Figure 1E), has garnered substantial attention as a promising therapeutic target for enhancing cognitive abilities. It is a wake-promoting agent that was approved in 1998 by the United States Food and Drug Administration (FDA) for treating daytime sleepiness in shift work sleep disorder or narcolepsy and obstructive sleep apnea [16]. Modafinil has been also extensively investigated as a cognitive enhancer in healthy volunteers [17] and in the therapy of neuropsychiatric disorders, where cognitive deficit is a core and disabling symptom, such as in schizophrenia [18,19], attention deficit hyperactivity disorder [20,21], depression [22,23,24], and mood disorders [23,25]. This wake-promoting agent is also known in the literature as an atypical DAT inhibitor because of its unique binding mode, which can be differentiated to that of cocaine, and different pharmacological profile compared to amphetamine and amphetamine-like psychostimulants [26,27]. Both of modafinil’s enantiomers bind to the DAT and inhibit the reuptake of DA from the synaptic cleft into the neuron less potently than cocaine, with *R*-modafinil having an approximately 3-fold higher affinity than *S*-modafinil [27]. However, modafinil has also low specificity to serotonin (SERT) and the norepinephrine (NET) transporters, which may lead to unwanted additional CNS effects, such as headache, nausea, rhinitis, nervousness, diarrhea, back pain, anxiety, dizziness, dyspepsia, and insomnia [17,28].

Research on modafinil analogs has gained significant momentum in recent decades due to the compound’s unique pharmacological properties and potential therapeutic applications [29,30,31,32,33,34]. The primary objectives of these studies were to identify modafinil analogs that can address a wide range of cognitive deficits and neurological conditions effectively, with a reduced risk of side effects. In our previous study, we reported a series of modafinil analogs with higher DA reuptake inhibition activity and higher selectivity for the DAT in comparison to modafinil [33]. These improvements were achieved by making strategic modifications involving the substitution of the carboxamide moiety with five- and six-membered aromatic heterocycles [33]. The thiazole-containing analog CE-123 (5-[(Diphenylmethyl)sulfinyl]thiazole, Figure 1H) has been shown to enhance the cognitive flexibility in male Sprague-Dawley rats without producing impulsive responding compared to *R*-modafinil and to enhance memory acquisition and memory retrieval at doses that did not change the locomotor activity of the animals [35,36,37]. In reuptake inhibition assays using HEK293 cells stably expressing human isoforms of the DAT, *S*-CE-123 demonstrated an almost 8-fold higher in vitro activity on the DAT than its *R*-enantiomer [35]. In addition, Kristofova et al. (2018) have used the mouse cell line cerebEND as an in vitro model to study the ability of CE-123 to cross an in vitro model of the blood–brain barrier (BBB) [36]. The results showed that CE-123 could penetrate the BBB in a similar manner to diazepam, and as previously observed for modafinil [36,38]. In addition, Kristofova et al. (2018) also quantified *S*-CE-123 and *R*-modafinil in rat plasma, cerebrospinal fluid (CSF), and brains after a single intraperitoneal (IP) administration of *S*-CE-123 and *R*-modafinil at 10 mg/kg to Sprague-Dawley rats [36]. Yet, due to significant differences in the plasma pharmacokinetic (PK) profiles, governed primarily by differences in metabolism, direct comparison between two compounds after single IP dosing could be misleading.

The neuropharmacokinetic (neuroPK) parameters of the novel DAT inhibitor as well as modafinil have so been far assessed based on the total drug concentration in the plasma and the entire brain, usually at a single time point in a non-dedicated PK study. Yet, the total brain-to-plasma concentration ratio (K_p,brain_) is just one of the neuroPK parameters with a limited use for characterization of the extent of BBB transport [39,40]. According to the Free Drug Theory, the concentration of unbound (free) drugs, particularly at a site of action like the brain, governs the pharmacological effect(s), emphasizing the significance of unbound drug concentrations in distribution processes [41]. To this end, the unbound brain-to-plasma concentration ratio, K_p,uu,brain_, is the key neuroPK parameter to obtain for the assessment of the extent of BBB transport. With the Combinatory Mapping Approach (CMA) [42], evaluation of the neuroPK parameters of unbound drugs can be obtained in a more high-throughput mode, which makes it useful for CNS drug discovery [42]. The CMA employs several in vivo and in vitro methods to provide a comprehensive assessment of drug properties concerning BBB transport and intra-brain distribution. In an in vivo neuroPK study, the total concentrations of drugs in the brain and plasma are determined at the steady state, which is pivotal for assessing K_p,brain_ (Figure 1(A1)). In parallel, in vitro investigations involve the determination of unbound fractions of drugs in the plasma (f_u,plasma_) and brain tissue binding properties (f_u,brain_) using equilibrium dialysis and the brain slice method (V_u,brain_) (Figure 1(B1,C1)) [42,43,44,45,46,47]. Finally, a combination of these acquired parameters enables the estimation of K_p,uu,brain_ and the unbound drug cell partitioning coefficient (K_p,uu,cell_). K_p,uu,brain_ represents the ratio of brain interstitial fluid (ISF) to the plasma unbound drug concentrations at the steady state, providing a quantitative description of the net BBB drug transport. This parameter relies on three compound-specific parameters, K_p,brain_, V_u,brain_, and f_u,plasma_. Values closer to unity describe mainly passive transport at the BBB or reflect similar efflux and influx clearances, while values smaller than unity indicate a predominantly active efflux, and values higher than unity indicate potential active uptake. K_p,uu,cell_ represents the steady-state ratio of intracellular to extracellular unbound drug concentrations, describing the drug transport across the cellular membrane. Fridén et al. (2007) suggested the determination of K_p,uu,cell_ by combining V_u,brain_ and f_u,brain_ [48]. Since cellular barriers may exhibit asymmetries like those encountered at the BBB, the interpretation of K_p,uu,cell_ follows the same pattern as K_p,uu,brain_, with values either exceeding or falling below unity. For the purposes of this study, the CMA has been employed, offering a comprehensive evaluation of the neuroPK parameters of *S*-CE-123 and *R*-modafinil.

Liquid chromatography–high resolution mass spectrometry (LC–HRMS) is often applied for simultaneous quantification of small molecules and selective metabolite detection in PK/neuroPK studies. In general, the quantification of small-molecule drugs in tissues is challenging, especially in the brain tissue due to its unique matrix composition. A high lipid composition represents the major challenge for sample preparation development [49]. Another issue is the buffers used in experiments for in vitro screening, having a high salt composition as well as endogenous compounds, which may lead to unwanted matrix effects [49,50]. Therefore, when developing an LC–HRMS method for PK/neuroPK studies, the sample preparation represents a critical step in the method development.

In this context, this is the first study aimed at elucidating the tissue distribution profile, with a specific emphasis on CNS distribution and metabolic characteristics, of *S*-CE-123 in direct comparison to *R*-modafinil (Figure 1). We introduce a simple and robust LC–HRMS method suitable for the investigation of PK and neuroPK parameters related to *S*-CE-123 and *R*-modafinil. We demonstrate that there are distinct differences between *S*-CE-123 and *R*-modafinil within the context of CNS distribution. These distinctions encompass their respective abilities to cross critical CNS barriers, including the BBB, blood–spinal cord barrier (BSCB), and brain parenchymal cell membranes. Furthermore, our study investigates their metabolic stabilities, highlighting significant variations in the hepatic metabolism in human liver microsomes (HLMs) and rat plasma stability.

## 2. Results

### 2.1. Analytical Method

**Figure 2:**
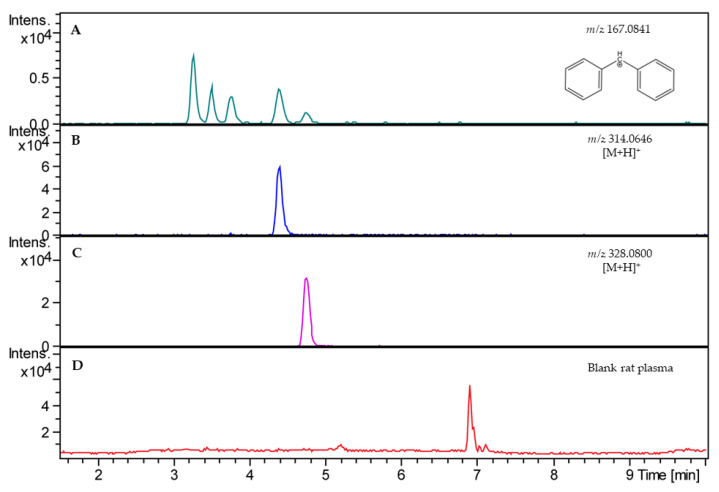
Extracted ion chromatograms (EICs) of blank rat plasma spiked with analytes at 500 ng/mL (**A**–**C**) compared with total ion chromatogram of blank rat plasma without analytes (**D**). In (**A**), an EIC of *m*/*z* 167.0841 for *R*-modafinil (RT = 3.3 min), modafinic acid (RT = 3.6 min), and modafinil sulfone (RT = 3.9 min). In (**B**), EIC of *S*-CE-123 (*m*/*z* 314.0646, RT = 4.5 min). In (**C**), EIC of IS (*m*/*z* 328.0800, RT = 4.8 min). RT—retention time.

The first step in method development included the analysis of the mass spectra of *R*-modafinil, its metabolites, *S*-CE-123, and CE-137, which was used as the internal standard (IS). The HRMS spectra were recorded in the range of mass-to-charge ratios (*m*/*z*) of 50–1550 in the positive ion mode. The sum formulas were determined based on the mass accuracy (Δ*m*/*z* ≤ 2 ppm) and isotopic pattern matching (SmartFormula algorithm). The analysis of the HRMS spectra showed that *R*-modafinil and its metabolites had a high abundant ion at *m*/*z* 167.0841, which corresponds to cleavage of stable secondary aromatic carbocation from the parent molecule (Figure 1A). Additionally, low abundant ions for *R*-modafinil at *m*/*z* 297.08 and 274.08 were also observed, corresponding to [M + Na]^+^ and [M + H]^+^, respectively. These low abundant ions were used for qualitative purposes. In the case of *S*-CE-123 and CE-137, both analytes showed protonated forms with highly abundant ions at *m*/*z* 314.0646 and 328.0800. Due to their structural similarities to *R*-modafinil, *S*-CE-123 and CE-137 also showed a diphenylmethyl cation at *m*/*z* 167.0841, though with low abundance. For quantification purposes, it was decided based on the ion abundance of each analyte to use extracted ion chromatograms (EICs) of *m*/*z* 167.0841 for *R*-modafinil, *m*/*z* 314.0646 for *S*-CE-123, and *m*/*z* 328.0800 for the IS. To obtain chromatograms with a suitable peak shape, higher sensitivity, and shorter running time, different ultra-high performance liquid chromatography (UHPLC) parameters were tested. The primary objective of the HPLC separation was to effectively separate *R*-modafinil from its metabolites, modafinic acid and modafinil sulfone, since all three analytes had a high abundant ion at *m*/*z* 167.0841. Overall, chromatographic separation was improved by using acetonitrile (ACN) instead of methanol (MeOH), due to an adequate peak shape and faster gradient elution. The addition of 0.1% formic acid (FA) to the water improved the formation of the *m*/*z* 167.0841 and lowered the abundance of [M + Na]^+^ in the case of *R*-modafinil, resulting in higher sensitivity. Figure 2 illustrates a representative LC–HRMS chromatogram of the analyte mixture containing *R*-modafinil, modafinic acid, modafinil sulfone, *S*-CE-123, and the IS. Notably, the method accomplished baseline separation of the analytes within 12 min, achieving an acceptable peak resolution (Rs), particularly between *R*-modafinil and its metabolite with an Rs of approximately 1.7.

### 2.2. Validation of Analytical Method

The LC–HRMS method was successfully partially validated in-house, and the results are presented in Table 1. The validation studies were designed in two blank matrices, rat plasma and 1:9 rat whole brain homogenate in phosphate-buffered saline (PBS), pH 7.4 (*w*:*v*). The validation parameters were determined at three levels of low, medium, and high (50, 500, and 1000 ng/mL). Each concentration was prepared in triplicate and measured three times. Analysis of the blank pooled matrix showed no potential endogenous matrix compounds at the retention times of the analytes and the IS (Figure 1D). For assessment of the generated data from the calibration curves, linear regressions was used, not forced through the origin, and no weights were applied. The calibration curves for both analytes ranged from 20 to 2000 ng/mL in each matrix, and each LC–HRMS run exhibited linearity with R^2^ > 0.99. The IS response in the blank matrix did not exceed ±5% of the average IS responses of the calibration standards and quality controls (QCs). Furthermore, the mean values for accuracy are reported in Table 1. In general, the presented analytical method is considered accurate as the reported values in both matrices and for both analytes were below ±15% [51,52]. The repeatability of the analytes in the rat plasma and brain homogenate ranged from 3.9 to 10.2% and from 2.4 to 8.6%, respectively. The intermediate precision, determined on three different days, ranged from 5.5 to 12.9% in the rat plasma and from 2.9 to 12.5% in the rat brain homogenate. According to the data shown in Table 1, the recoveries of the analytes in the rat plasma and brain homogenate ranged from 91 to 112% and 96 to 109%, respectively. In the case of the matrix effect, a consistent level of matrix effect was observed in both types of sample matrices and for all analytes, resulting in ion suppression as caused by the co-eluting impurities that competed with the analytes for ionization. The LODs for both analytes in the rat plasma and brain homogenate were 10 ng/mL, whereas the LOQs were 20 ng/mL in the rat plasma.

The important part of the method validation included testing of the stability of *R*-modafinil, particularly since its possible esterase/amidase-catalyzed degradation into modafinic acid in the rat plasma was hypothesized [53] and later confirmed in this study. The in vitro plasma stability of *R*-modafinil was tested in rat plasma as well as rat plasma containing 10% dimethylformamide (DMF) as an enzyme inhibitor, and the results are reported in Supplementary Materials Figure S1. During a 4 h incubation at 37 °C, the *R*-modafinil levels decreased to about 50%. Notably, modafinic acid was detected at the 2 and 4 h time points. However, in the rat plasma containing 10% DMF, the *R*-modafinil concentration remained stable, with values ranging from 96 to 98% of the initial concentration. In addition, the assessment of specificity has shown that the presence of modafinic acid did not affect the accuracy of the QCs (Supplementary Materials Table S1).

### 2.3. Metabolism Assays

**Figure 3:**
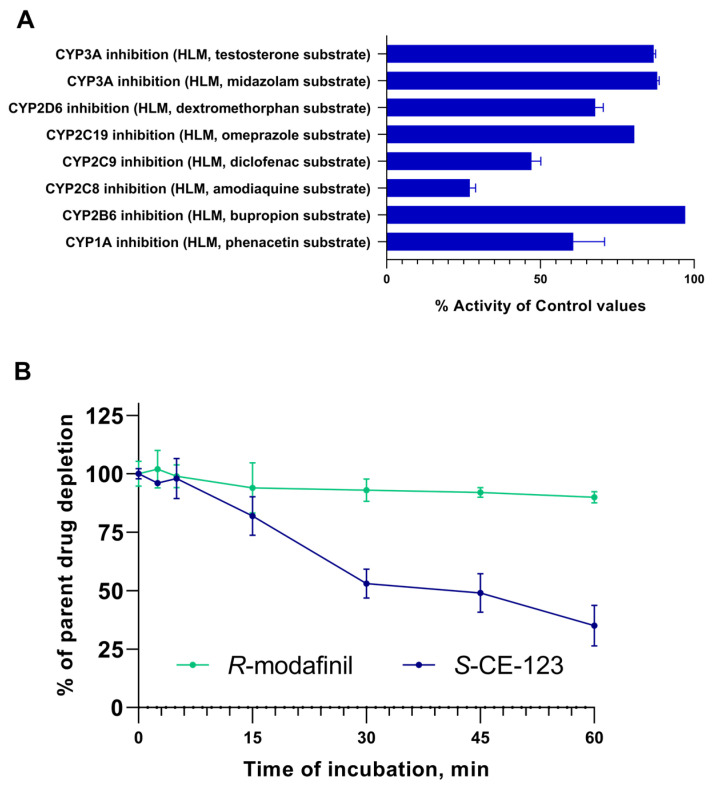
Inhibitory potential of *S*-CE-123 (10 µM) on eight CYPP450 isoforms (**A**) and metabolic stability curves of *S*-CE-123 and *R*-modafinil after 60 min incubation of test compound at 1 µM in HLMs (**B**) (n = 3, mean ± SD).

The inhibitory potential of *S*-CE-123 on eight cytochrome (CYP) P450 isoforms was examined using human liver microsomes (HLMs) (Figure 3A). The results revealed the most significant inhibition of control values in the CYP2C8 isoform at 27%. Moderate inhibitory effects were observed in the isoforms CYP2C9, CYP1A and CYP2D6, with values ranging from 47 to 68%. Low inhibition of the control values was found in the isoforms CYP2C19, CYP3A, and CYP2B6.

The results of in vitro HLM incubation of the test compound at 1 µM, expressed as % of the parent drug remaining, are shown in Figure 3B. These in vitro metabolic stability experiments indicated that after 60 min of incubation, only 35% of the *S*-CE-123 remained, indicating a significant metabolism. In contrast, *R*-modafinil exhibited a notably high metabolic stability, with 90% remaining. The high metabolic stability of *R*-modafinil in the HLMs could indicate that the liver may not be its primary elimination site. Furthermore, incubation of *R*-modafinil at concentrations of 5, 10, and 50 µM yielded similar outcomes, with ≤10% being metabolized. However, the formation of the metabolite modafinil sulfone was observed at all concentrations during incubation, whereas modafinic acid was not detected. The values obtained for half-life (t_1/2_) and intrinsic clearance (CL_int_) were found to be 39 min and 3.6 µL/min/mg for *S*-CE-123 and 364 min and 0.4 µL/min/mg for *R*-modafinil.

The incubation of *S*-CE-123 with HLMs at 50 µM and processing of the obtained LC–HRMS raw data using MZmine 3.4.27 identified a potential metabolite, M1. The characteristic molecular ion of M1 was found at *m*/*z* 330.0624 with a retention time of 5.5 min. This corresponds to the addition of 16 Da to protonated *S*-CE-123, suggesting that M1 likely is formed from the hydroxylation of *S*-CE-123, probably on one of its two aromatic moieties. However, the exact hydroxylation site was not yet confirmed. The sum formula of M1 was determined based on the mass accuracy (Δ*m*/*z* ≤ 2 ppm) and isotopic pattern matching by using the SmartFormula algorithm from the software Compass DataAnalysis (Bruker Daltonics, Billerica, MA, USA, version 4.2) and was found to be C_17_H_16_NO_2_S_2_ (Supplementary Materials Figure S2).

### 2.4. Biodistribution

**Figure 4:**
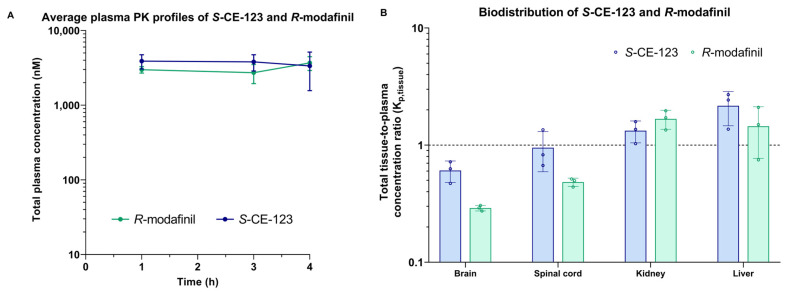
Semilogarithmic total plasma concentration–time profiles (**A**) and tissue distribution (**B**) of *S*-CE-123 and *R*-modafinil in rats (n = three per compound) after a 4 h intravenous constant infusion at the dosage of 20 mg/kg.

The tissue distribution of the total *S*-CE-123 and *R*-modafinil in the rat plasma, brain, spinal cord, CSF, liver, and kidneys was studied after a 4 h intravenous constant infusion at a dosage of 20 mg/kg in Sprague-Dawley rats (n = three rats per compound). The results are summarized in Figure 4 and Table S2 within Supplementary Materials. As demonstrated in Figure 4A, a steady state was achieved in the plasma for both compounds, reaching an approximately 3000 nM (ca. 1000 ng/mL) targeted concentration.

To assess the extent of the tissue distribution, the total tissue-to-plasma concentration ratio (K_p,tissue_) was calculated. Notably, both test compounds exhibited the highest K_p,tissue_ values in the liver and kidneys, while the lowest were observed in the brain. The order of K_p,tissue_ from highest to lowest for *S*-CE-123 was liver > kidneys > spinal cord > brain, and for *R*-modafinil, it was kidneys > liver > spinal cord > brain. Moreover, the metabolite of *S*-CE-123 M1 was detected in all samples, with the highest signals observed in the liver and kidneys, and the lowest in the CSF and brain (Supplementary Materials in Figure S3). The metabolites of *R*-modafinil, modafinic acid and modafinil sulfone, were detected together in the plasma, CSF, liver, and kidneys (Supplementary Materials in Figure S3). Surprisingly, modafinil sulfone was also detected in the rat brain and spinal cord tissue, as shown in Figure S4 of Supplementary Materials. However, it is worth noting that after the integration of the modafinil sulfone peak, the S/N ratio was below the LOQ (S/N < 10, though above the LOD, S/N > 3), rendering it non-quantifiable. Modafinic acid had the highest signals in the plasma and kidneys and modafinil sulfone in the liver and kidney tissue.

### 2.5. Unbound Drug PK Parameters

**Figure 5:**
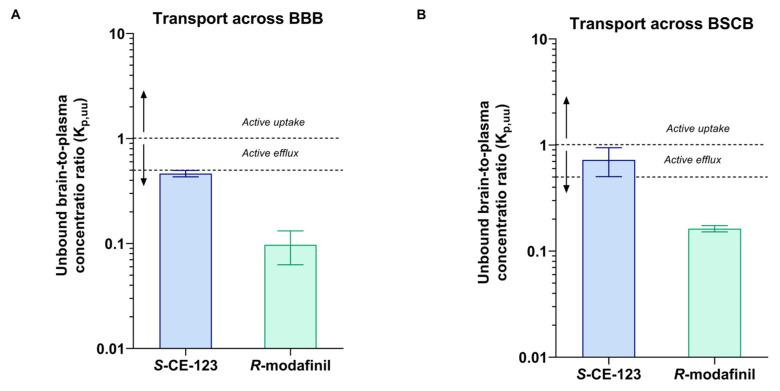
Unbound brain/spinal-cord-to-plasma concentration ratio K_p,uu_ of *S*-CE-123 and *R*-modafinil characterizing the extent of transport across the blood–brain barrier (**A**) and across the blood–spinal cord barrier (BSCB), (**B**) in rats (n = three per compound). Dotted lines represent K_p,uu_ at 1 and 0.5. Data presented as mean and standard deviation estimated using uncertainty propagation method [54].

As K_p,brain_ is a very complex parameter involving binding to plasma proteins, as well as the binding and uptake to the brain tissue, the extent of BBB transport of *S*-CE-123 and *R*-modafinil was assessed using K_p,uu,brain_ (Equation (4)). The values obtained were 0.46 and 0.097, respectively (Figure 5 and Table 2). These results demonstrate that both drugs are primarily subject to net active efflux transport from the brain, more profound for *R*-modafinil. Remarkably, when it comes to the BSCB transport, *S*-CE-123 showed K_p,uu_ values closer to unity, indicating the possibility of passive diffusion at the BSCB (Figure 5B). On the other side, *R*-modafinil showed rather similar performance at both the BSCB and BBB, with a dominating net active efflux (Figure 5). Table S2 in Supplementary Materials provides an overview of the total CSF concentrations. However, due to the challenges associated with collecting the CSF from the cisterna magna in rats, successful CSF sampling was not achieved for all three subjects. Specifically, CSF samples from only two rats were available for the analysis of *S*-CE-123, while data regarding *R*-modafinil in CSF were obtained from a single rat. Consequently, the total CSF concentrations for *S*-CE-123 were 570 and 104 ng/mL, while *R*-modafinil displayed a concentration of 784 ng/mL.

Equilibrium dialysis was employed to assess the plasma protein binding. The metabolism of *R*-modafinil into modafinic acid occurred in vitro after spiking blank rat plasma with *R*-modafinil and 10% DMF as an inhibitor. The biotransformation was rapid, occurring during the spiking stage before equilibrium dialysis started, with an *R*-modafinil-to -modafinic-acid peak area ratio of 88:12%. After 5 h of equilibrium dialysis, this ratio shifted to 76:24%. In spite of this, the stability of both compounds during 5 h incubation was within the acceptable range of 100 ± 30%, i.e., not compromising the obtained results. It was beyond the scope of this work to further study the *R*-modafinil plasma instability. The f_u,plasma_ mean values were 0.252 and 0.797, meaning that 25% and 80% of *S*-CE-123 and *R*-modafinil, respectively, are free (unbound) in plasma. Respectively, the mean unbound concentration in plasma (C_u,plasma_) in the in vivo study was determined to be 840 nM for *S*-CE-123 and 2912 nM for *R*-modafinil (Supplementary Materials in Table S3). In addition, using Equation (6), the in vivo mean unbound concentration of *S*-CE-123 in the brain ISF (C_u,brainISF_) was found to be 386 nM under steady-state conditions, while for *R*-modafinil, it was 282 nM (Supplementary Materials in Table S3).

The assessment of brain tissue binding using rat brain homogenate revealed that both drugs bind to the brain parenchymal tissue, with mean f_u,brain_ values of 0.035 for *S*-CE-123 and 0.23 for *R*-modafinil. Notably, *S*-CE-123 exhibits an extensive brain tissue binding capacity, which is 6.6-fold higher compared to that of *R*-modafinil (Table 2). The unbound volume of distribution (V_u,brain_), measured using in vitro brain slice assay, was 5.21 and 3.73 mL/g of brain for *S*-CE-123 and *R*-modafinil, respectively (Table 2).

The estimation of the extent of brain parenchymal cellular barrier transport was performed by means of K_p,uu,cell_ (Equation (5)). The results for *S*-CE-123 and *R*-modafinil were 0.18 and 0.85, respectively. This indicates that *S*-CE-123 will primarily reside in the brain interstitial space, while *R*-modafinil has a rather equal distribution across both sides of the plasma membrane of the brain parenchymal cells.

To facilitate interpretation, a simulation of the unbound plasma and unbound brain concentration–time profiles was performed based on the obtained parameters of the rate and extent of BBB transport, as well as the extent of intra-brain distribution, using a simple structural model, as outlined in Supplementary Figure S5 [36,38]. Remarkably, at rather similar steady-state total plasma concentrations (3400 nM for *S*-CE-123 and 3800 nM for *R*-modafinil), the unbound brain steady-state concentrations were 1.3-fold different, i.e., ca. 400 nM for *S*-CE-123 and 300 nM for *R*-modafinil, in spite of significant ca. 5-fold differences in K_p,uu,brain,_ (Supplementary Figure S6).

## 3. Discussion

Modafinil, particularly its *R*-enantiomer (*R*-modafinil), and its novel analog, *S*-CE-123, have been explored as potential neurotherapeutics for the treatment of cognitive decline. In this study, we developed an LC–HRMS method tailored to investigate the tissue distribution profile of these compounds, with a specific focus on CNS distribution and metabolism in the HLMs. For the first time, we show that there are distinct differences between *S*-CE-123 and *R*-modafinil in the context of unbound compound CNS distribution in rats. Our findings highlight that *S*-CE-123 crosses the BBB to a greater extent than *R*-modafinil, leading to a higher unbound concentration in the brain ISF when administered at a similar dose. We also demonstrate an increased presence of *S*-CE-123 at the target brain interstitial site by means of K_p,uu,cell_. In addition, we show that there are significant variations in their metabolic stability, with *S*-CE-123 having a 9.3-fold faster metabolism in HLMs and *R*-modafinil undergoing metabolism in rat plasma.

At the early stages of drug discovery and development, neuroPK studies are crucial for determining the expected concentration of a compound within the targeted brain area and for comparing this to systemic drug exposure and pharmacodynamic (PD) readouts. As a result, the assessment of K_p,uu,brain_ is fundamental for determining how effectively a drug can cross the BBB without being influenced by plasma protein or brain tissue binding [39,42,55]. K_p,uu,brain_ is a key parameter that can be derived using the well-established CMA, which also offers a comprehensive evaluation of other neuroPK parameters such as V_u,brain_, f_u,brain_, and K_p,uu,cell_ [42]. In the present study, we have demonstrated that *S*-CE-123 and *R*-modafinil predominantly exhibit net active efflux transport from the brain, which is more profound for *R*-modafinil (K_p,uu,brain_, Table 2). With its more effective transport across the BBB in rats, *S*-CE-123 exhibited a higher unbound concentration in the brain ISF compared to *R*-modafinil. Moreover, *S*-CE-123 also has reported increased selectivity and affinity for the DAT relative to *R*-modafinil, with an inhibitory constant (K_i_) of 610 nM compared to 780 nM for *R*-modafinil in cell lines expressing human DAT [27,56]. While other DAT modulators (e.g., methylphenidate, amphetamine, or methylphenidate) have higher or similar DAT affinities, with K_i_ values ranging from 60 to 640 nM in cell lines expressing human DAT [57], *S*-CE-123 has a negligible activity on SERT and NET [36].

Moreover, our findings also indicate that *R*-modafinil displays instability in rat plasma both in vivo and in vitro, with 50% being metabolized after 4 h at 37 °C, yielding its metabolite, modafinic acid. The amide moiety in modafinil makes it prone to hydrolysis by enzymes such as esterases and/or amidases, resulting in modafinic acid, which does not seem to have any significant activity in the brain or periphery [16,58,59]. Differences in the activities of esterase/amidase enzymes can influence a drug’s plasma stability, which varies across species and can be gender-specific. For instance, rodents have a notably higher hydrolytic rate than dogs, while humans demonstrate the slowest rate [60]. This phenomenon must be taken into account not only in PK studies of modafinil in rats but importantly in PD experiments. It should also be considered that 80% of the free unbound *R*-modafinil in rat plasma is bioavailable for transport into the brain. However, not the entire fraction of the free compound in the plasma is able to cross the BBB, requiring measurement of K_p,uu,brian_ and not making any conclusions on BBB transport based on assessment of the fraction of the unbound drug in the plasma. Various mechanisms at the BBB, including efflux transporters primarily belonging to the ATP-binding cassette (ABC) family such as P-glycoprotein (P-gp) and breast-cancer-resistant protein (BCRP), govern drug delivery to the brain parenchyma. Indeed, an in vitro study has shown that both modafinil enantiomers are not only substrates of P-gp but also weak inhibitors of this efflux transporter [61]. The clinical implications remain unidentified.

Furthermore, once the drug enters the brain, it may be distributed intracellularly, governed by various mechanisms, including nonspecific binding and specific binding to receptors like DA, SERT, NET, etc. [62]. Studies exploring nonspecific binding elucidate the extent of such binding in the brain compared to the unbound drug in the brain ISF (f_u,brain_ and V_u,brain_). The parameter f_u,brain_ primarily reflects intracellular nonspecific binding to the brain tissue, while V_u,brain_ represents the overall uptake into the brain tissue, including both pH partitioning and active uptake. By integrating these two parameters, the intracellular accumulation of unbound drugs can be estimated (K_p,uu,cell_), which reflects the steady-state ratio between intracellular and extracellular unbound drug concentrations [48,55]. In the present study, we have shown that upon entering the brain, *S*-CE-123 and *R*-modafinil distribute within the brain tissue, with *S*-CE-123 exhibiting an extensive brain tissue binding capacity (Table 2). *S*-CE-123 predominantly resides in the brain interstitial space, while *R*-modafinil is more likely to distribute equally across both sides of the plasma membrane of the brain parenchymal cells (K_p,uu,cell_, Table 2). Given that *S*-CE-123 is a known DAT inhibitor and that the DAT is located in the presynaptic plasma membrane, the observed values are desirable and suggest an increased presence of *S*-CE-123 at the target site.

During the early stages of drug discovery, a crucial aspect also involves understanding how compounds distribute across tissues and undergo metabolism. The tissue distribution profile of a potential drug determines which organs or tissues are exposed to the drug and its metabolites, thereby influencing both efficacy and potential toxicity [63]. Primarily taking place in the liver, metabolism impacts drug clearance, half-life, and the potential for drug–drug interactions [64]. CYP P450 enzymes play a pivotal role in drug metabolism, and understanding their interaction with candidate drugs is essential [65]. In the present study, we have demonstrated that *S*-CE-123 exhibited the most potent inhibition of the isoform CYP2C8, with moderate to low inhibition of the isoforms CYP2C9, CYP1A, CYP2D6, CYP2C19, CYP3A, and CYP2B6. The CYP2C8 isoform comprises 7% of the total CYP content in the liver and is responsible for the oxidation of about 5% of drugs cleared by phase I metabolism [66]. The typical substrate drugs of CYP2C8 include anticancer agents (paclitaxel, cabazitaxel, enzalutamide), antidiabetic agents (repaglinide, pioglitazone, rosiglitazone), antimalarial agents (amodiaquine, chloroquine), lipid-lowering agent (cerivastatin), and others [66,67]. CYP2C9 is responsible for the metabolism of approximately 10–20% of currently marketed drugs, including warfarin, losartan, fluvastatin, diclofenac, ibuprofen, etc. [66]. Additionally, CYP2D6 is also expressed in the brain, and its role in the metabolism of DA, noradrenalin, and serotonin should be considered [68,69,70,71]. In relation to modafinil, Robertson et al. (2000) have reported that modafinil modestly induces CYP1A2, CYP2B6, and CYP3A4/5, and shows reversible inhibition of CYP2C19 (in HLMs), as well as apparent suppression of CYP2C9 activity (in primary cell cultures of humane hepatocytes) [72].

In vitro metabolic stability studies have revealed that *S*-CE-123 is extensively metabolized by HLM enzymes compared to *R*-modafinil. *S*-CE-123 has been shown to undergo metabolic hydroxylation in the HLMs to form one metabolite, M1. The presence of this metabolite has also been confirmed in rat plasma after a 4 h intravenous constant infusion of *S*-CE-123 (20 mg/kg), with its formation being time-dependent (Supplementary Materials in Figure S7). Although *R*-modafinil has been rather slowly metabolized by HLM enzymes, the formation of modafinil sulfone has been observed. Modafinil undergoes oxidation mediated by CYP3A4 to produce modafinil sulfone [58]. Wong et al. (1999) have found that after a single administration of a 200 mg oral dose of modafinil, 35–60% of the dose was detected in the urine as modafinic acid, representing its major metabolite [73]. In a follow-up study, Wong et al. (1999) have shown that modafinil sulfone was found in the urine as a minor metabolite, suggesting that the results of HLM incubation are in correlation with the reported data [74].

In our tissue distribution studies, both compounds exhibited high K_p,tissue_ values in the liver and kidney samples. Notably, *S*-CE-123 had the highest K_p,tissue_ in all tissue samples, except in the kidneys, where *R*-modafinil dominated. Interestingly, the detection of M1, a metabolite of *S*-CE-123, and modafinil sulfone in the rat brain and spinal cord samples was unexpected. While modafinil sulfone has been reported to be inactive in regard to the receptors involved in the wake-promoting effect of modafinil [58,75], its presence in the CNS tissues should be considered in the evaluation of modafinil’s pharmacological activity. Some authors have suggested that modafinil and its metabolites, when administered alone or together with antiepileptic drugs, enhanced anticonvulsive activity in rodents [76,77]. This hints at the possibility that these metabolites, including modafinil sulfone, may exert modulatory effects on the CNS, potentially influencing the overall pharmacological profile of the parent compound. Understanding these relationships is important for a comprehensive evaluation of the PD of *S*-CE-123 and *R*-modafinil, along with their metabolites. Such insights could be crucial to their efficacy and safety profiles. Accordingly, further research is needed to resolve questions about the pharmacological activity of *R*-modafinil’s/*S*-CE-123’s metabolites.

## 4. Materials and Methods

### 4.1. Materials

The *R*-modafinil (purity 98.0%), modafinil sulfone (purity 96.0%), *S*-CE-123 (purity 99.7%), and CE-137 (purity 97.6%) were in-house synthesized according to already published protocols [33,34,35,78,79]. The modafinic acid analytical standard was purchased from Fluorochem Ltd. (Glossop, UK). The LC–MS-grade ACN, MeOH, and water were purchased from Merck (Darmstadt, Germany). The formic acid (FA) and phosphoric acid (PA) were supplied by Carl Roth (Karlsruhe, Germany). The HEPES (4-(2-hydroxyethyl)-1-piperazineethanesulfonic acid) and 2-hydroxypropyl-β-cyclodextrin (HPβCD) were purchased from Sigma-Aldrich (Steinheim, Germany). The sterile saline was purchased from Fresenius Kabi (Bad Homburg, Germany). The human liver microsomes (HLMs) and beta-nicotinamide adenine dinucleotide phosphate-reduced tetrasodium salt (NADPH) were purchased from Thermo Fisher Scientific (Vienna, Austria). The dipotassium phosphate and potassium dihydrogen phosphate, used for the preparation of phosphate buffer, pH = 7.4, were purchased from Acros Organics (Geel, Belgium).

### 4.2. Preparation of Stock Solutions and Internal Standard Solutions

The stock solutions were prepared in ACN at a concentration of 1 mg/mL or in the case of HLM incubation at 0.01 M and stored in HPLC vials at −20 °C. The internal standard (IS) solution contained the modafinil analog CE-137 at a concentration of 500 ng/mL or, in the case of HLM incubation, 1 µM.

### 4.3. Animals

Experiments were performed on 14 drug-naïve male 250–300 g Sprague Dawley rats (Taconic, Lille Skensved, Denmark) in accordance with guidelines from Swedish National Board for Laboratory Animals and were approved by the Animal Ethics Committee of Uppsala, Sweden (ethical approval Dnr 5.8.18-12230/2019). All rats were housed in groups at 20 to 22 °C under a 12 h light/dark cycle with ad libitum access to food and water.

### 4.4. In Vitro Drug Tissue Binding Assay

Equilibrium dialysis was used to assess the fractions of unbound *S*-CE-123 and *R*-modafinil in the rat plasma (f_u,plasma_) and in the rat whole brain homogenate (f_u,brain_) by evaluating the plasma protein binding (PPB) and brain tissue binding (BTB) obtained. A Teflon 96-well plate (model HTD96b, HTDialysis LLC, Gales Ferry, CT, USA) was used in all tissue-binding experiments. Th evaluation of PPB was determined according to van Liempd et al. (2011) [45] and BTB according to the previously published protocols [42,46,80]. Briefly, the undiluted plasma (N = 3, n = 3 per compound) was spiked with either *S*-CE-123 and *R*-modafinil to final concentrations of 10 µM. To inhibit the metabolization of *R*-modafinil to modafinic acid [53], the plasma contained 10% DMF. The whole brain tissue from three rats was individually homogenized in 9 volumes of phosphate-buffered saline (PBS), pH 7.4. The brain tissue homogenates were spiked with either *S*-CE-123 or *R*-modafinil to final concentrations of 10 µM. Then, 100 µL of spiked plasma or brain tissue homogenate in triplicate was dialyzed against an equal volume of PBS, pH 7.4, for 5 h at 37 °C and 200 rpm. Furthermore, 50 µL was taken from both sides of the membrane at the end of incubation. The unbound fraction of the compounds in the plasma (f_u,plasma_) or in the diluted (D) brain homogenate (f_u,hD_) was calculated according to the following equation:

(1) $$f_{u,plasma}\;or\;f_{u,hD}=\frac{C_{buffer}}{C_{tissue}}$$

where C_buffer_ represents the concentration of the compound measured in the buffer and C_tissue_ is the concentration measured in the plasma or brain tissue homogenate. Due to dilution, f_u,hD_ is usually higher than the actual f_u,brain_. Thus, f_u,hD_ was corrected for the dilution factor (D), which is in this case 10 times.

(2) $$f_{u,brain}=\frac{f_{u,hD}}{{D+f}_{u,hD}-D\cdot f_{u,hD}}$$

### 4.5. Brain Slice Assay 

The volume of the distribution of unbound *S*-CE-123 and *R*-modafinil in the brain (V_u,brain_, mL·g brain^−1^) was estimated using the brain slice method according to the published protocols [43,44]. Briefly, six 300 µm brain slices were cut from one rat brain (n = 3 rats) using a vibrating-blade microtome Leica VT1200 (Leica Microsystems AB, Stockholm, Sweden), and incubated in HEPES-buffered artificial extracellular fluid (aECF) containing *S*-CE-123 and *R*-modafinil with an initial concentration of 200 nM for 5 h at 37 °C, a rotation speed of 45 rpm, and a constant oxygen flow. The buffer and brain slices were sampled at the end of the incubation. The slices were individually weighed and homogenized in 9 volumes (*w*:*v*) of aECF. Assuming that at equilibrium, the concentration of the drug in the aECF was equal to the interstitial fluid concentration in the brain slice, V_u,brain_ was estimated from Equation (3) as the ratio of the amount of the drug in the entire brain slice (A_brain_, nmole·g brain^−1^) to the measured final aECF concentration (C_buffer_, nmole·mL^−1^).

(3) $$V_{u,brain}=\frac{A_{brain}-V_{i}\cdot C_{buffer}}{C_{buffer}\cdot (1-V_{i})}$$

where V_i_ (mL·g brain^−1^) is the volume of the surrounding brain slice layer of the aECF. A volume V_i_ of 0.094 mL·g brain^−1^ was used in the calculations, as obtained using [^14^C] inulin as the marker [43].

### 4.6. Tissue Biodistribution Studies

In order to minimize the usage of animals and gain high confidence in the tissue distribution, a 4 h constant intravenous infusion study was designed based on the systemic pharmacokinetic parameters determined elsewhere. The catheters were surgically implanted a day before the PK experiment. The rats (n = 3 per compound) were then individually placed into a CMA 120 system (CMA, Solna, Sweden). *S*-CE-123 and *R*-modafinil were administered via a femoral vein catheter in 10% 2-hydroxypropyl-β-cyclodextrin (HPbCD) in 0.9% saline as a 4 h intravenous constant infusion. A dose of 20 mg/kg was administered as a combination of an initial short 10 min infusion as a loading dose followed by a constant infusion up to 4 h, targeting a steady-state total plasma concentration of around 1000 ng/mL for each compound (based on the pilot study results). Blood was collected into heparinized Eppendorf tubes via a femoral artery catheter at 0 (before infusion starts) 1, 2, 3, and 4 h after the infusion started and immediately centrifuged at 10,000 rpm for 5 min at +4 °C. Plasma was then collected and kept at −20 °C. At 4 h, the rats were anesthetized via inhalation of 2.5% isoflurane (Abbot Scandinavia, Solna, Sweden), balanced with 1.5 L/min oxygen, after which the terminal blood and tissue samples were collected. The terminal CSF was collected from the cisterna magna (n = 2 for *S*-CE-123, n = 1 for *R*-modafinil). The tissue samples (brain, spinal cord, liver, and kidney) were rapidly removed, pre-weighed into a 2 mL hard tissue homogenizing tube with mini-beads, and subsequently homogenized in 2 volumes of MQ water using a 4-Place Bead Mill Homogenizer (VWR, Stockholm, Sweden) at the maximal speed for 2 min and thereafter kept at +4 °C. The plasma and tissue homogenate samples were stored at −20 °C until LC–MS analysis.

### 4.7. NeuroPK Parameters

The unbound brain-to-plasma concentration ratio, K_p,uu,brain_, was calculated by using the three compound-specific parameters: K_p,brain_, V_u,brain_, and f_u,plasma_ [42]:

(4) $$K_{p,uu,brain}=\frac{K_{p,brain}}{V_{u,brain}\cdot f_{u,plasma}}$$

K_p,uu,brain_ values closer to unity describe a mainly passive transport at the BBB or reflect similar efflux and influx clearances, while values smaller than unity indicate a predominantly net active efflux, and values higher than unity indicate potential active uptake.

The unbound intracellular-to-extracellular (interstitial) concentration ratio, K_p,uu,cell_, was estimated by using the V_u,brain_ and f_u,brain_ parameters [42]:

(5) $$K_{p,uu,cell}=V_{u,brain}\cdot f_{u,brain}$$

Due to the multiparameter character of K_p,uu,brain_ and K_p,uu,cell_ assessment (Equations (4) and (5)), the estimation of variability around the mean values was calculated using an error propagation method as described in Loryan et al. (2017) [54].

The K_p,uu,brain_ values were further used to calculate the unbound drug concentration in the brain interstitial fluid C_u,brain,ISF_, according to Loryan et al. (2014) [42]:

(6) $$C_{u,brain,ISF}=K_{p,uu,brain}\cdot C_{u,plasma}$$

In order to present the unbound plasma and brain concentration profiles of *S*-CE-123 and *R*-modafinil, a simulation exercise was performed using Berkeley Madonna (version 8.3.18 for Windows, Berkeley, CA, USA). The simulation was performed at a given initial total plasma concentration to be equal to the steady-state concentrations obtained in the neuroPK study, utilizing the parameters listed in Supplementary Table S4. A two-compartment model consisting of blood and brain compartments with a focus on the unbound concentrations was used for the simulation exercise (see details on the structure of the model in Supplementary Figure S5). The representative unbound plasma and brain profiles are presented in Supplementary Figure S6.

### 4.8. Drug Metabolism

In vitro metabolism assays for the cytochrome (CYP) P450 inhibition of *S*-CE-123 was outsourced to Eurofins Panlabs (St. Charles, MO, USA, study number 100050638). Briefly, 0.1 mg/mL of the HLMs was incubated for 10 min at 37 °C with substrate (10 µM phenacetin for CYP1A, 100 µM bupropion for CYP2B6, 10 µM amodiaquine for CYP2C8, 10 µM diclofenac for CYP2C9, 0.5 µM omeprazole for CYP2C19, 5 µM dextromethorphan for CYP2D6, 5 µM midazolam for CYP3A, and 50 µM testosterone for CYP3A4) in the presence/absence of *S*-CE-123 (10 µM). The peak areas corresponding to the metabolite of each substrate were recorded. The percent of control activity was then calculated by comparing the peak area obtained in the presence of the test compound to that obtained in the absence of the test compound. Subsequently, the percent inhibition was calculated by subtracting the percent control activity from 100 for each compound. The IC_50_ values (concentration causing a half-maximal inhibition of control values) were determined using non-linear regression analysis of the concentration–response curve using Hill equation curve fitting.

An in vitro metabolism assay to study the phase I metabolism in the HLMs was in-house-developed. Optimization of the protocol included the test compound concentration (1, 5, 10 m and 50 µM) and extraction solvents (ACN and MeOH). In the optimized protocol reaction solution, consisting of 100 mM phosphate buffer (pH 7.4), 0.5 mg/mL of microsomal protein and 1 µM of *S*-CE-123 or *R*-modafinil was first pre-warmed for 5 min at 37 °C using the Thermomixer^®^ comfort (Eppendorf, Hamburg, Germany). The metabolic assay was initiated by the addition of 10 mM NADPH to the reaction solution and terminated after 0, 2.5, 5, 15, 30, 45, and 60 min via the addition of 200 µL of ice-cold MeOH containing the IS solution. The samples were than vortexed and spun for 15 min (14,000× *g*, 4 °C). The supernatant was collected, evaporated to dryness using the Concentrator plus (Eppendorf), and reconstituted with 100 µL of ACN/0.1% FA in water 10:90 (*v*:*v*). To ensure that the assay was performing adequately, the incubation of 1 µM diazepam at the same assay conditions as described was conducted, and the formation of its metabolites temazepam and nordazepam was monitored. A negative control included the absence of NADPH, the test compound, or HLMs during assay. The *S*-CE-123 or *R*-modafinil metabolic stability was expressed by calculating the percentage (%) of the parent drug remaining after incubation with the HLMs. The metabolic stability curves of the tested compounds were established by plotting the incubation time against the natural logarithm of the % parent drug remaining. The slope (k) of the linear part was utilized for the in vitro half-life (t_1/2_, min) as ln2/k. The V (µL/mg) was calculated as the volume of the reaction solution (100 µL) divided by the protein amount in the incubation (0.5 mg). The protein amount was predetermined by the supplier and the content was checked by using a Pierce™ BCA Protein Assay Kit (Thermo Fisher Scientific, Vienna, Austria) for each freshly opened tube. The intrinsic clearance (CL_int_, µL/min/mg) was calculated according to the following equation:

(7) $${CL}_{int}=\frac{0.693}{t_{1/2}}\cdot V$$

To study the metabolite formation of *S*-CE-123 using HLMs, a single high concentration (50 µM) was employed with the previously described protocol to achieve higher concentrations of metabolites for accurate detection. A non-targeted workflow was used with the metabolomics software MZmine 3.4.27, which allowed a comparison between the treated samples and the control samples [81]. The workflow is described in the Section 4.12.

### 4.9. Sample Preparation for Analysis

For all the rat plasma and CSF samples, an aliquot of 50 µL was spiked with 50 µL of 4% PA in water containing the IS solution to disrupt the drug–protein binding. For all rat tissue samples (brain, spinal cord, liver, and kidney), an aliquot of 50 µL was spiked with 50 µL ice-cold ACN containing the IS solution for protein precipitation. The samples were vortexed and spun down for 10 min (3000× *g*, 4 °C) and 25 µL of clear supernatant was diluted with 225 µL 0.1% FA in water to achieve 10% of ACN in the solution. SPE clean-up was performed using an Oasis PRiME HLB µElution 96-well plate, 3 mg (Waters Corporation, Vienna, Austria). The SPE cartridges were preconditioned and equilibrated with 1 × 200 µL ACN and 0.1% FA in water each. The samples were loaded onto the cartridges and washed with 2 × 200 µL 0.1% FA in water and 1 × 200 µL ACN/0.1% FA in water 10:90 (*v*:*v*). The analytes were eluted from the SPE cartridge into a collection plate with 2 × 25 µL ACN/0.1% FA in water 70:30 (*v*:*v*). All the samples were diluted with 50 µL of 0.1% FA in water and 10 µL of sample was injected onto the LC–HRMS system.

### 4.10. LC–HRMS Analysis

All samples, blanks, standards, and quality control samples in the respective matrices were analyzed on a UHPLC system (Dionex UltiMate 3000 RSLC series system, Thermo Fisher Scientific, Inc., Dreieich, Germany) coupled with a high-resolution mass spectrometer (maXis HD Q-TOF, Bruker Daltonics, Bremen, Germany) equipped with a heated electrospray ionization (ESI) source. Reversed-phase chromatographic separation was performed on a Kinetex Phenyl-Hexyl column (2.6 µm, 2.1 × 50 mm, Phenomenex, Torrance, CA, USA), equipped with a precolumn (2.6 µm, Phenomenex, Torrance, CA, USA), at a flow rate of 0.4 mL/min. Eluent A was water containing 0.1% FA and eluent B ACN. A multi-step gradient was optimized as follows: the initial mobile phase composition (5% eluent B) was held constant for 1 min, followed by a linear increase to 30% eluent B until minute 1.5, to 40% eluent B until minute 5.5, and to 95% eluent B until minute 6. After a hold time of 2 min, the column was re-equilibrated for 2.5 min at the initial conditions (5% eluent B). The column compartment and the autosampler were maintained at 40 °C and at 8 °C, respectively. The heated ESI interface was operated in positive mode at the following conditions: capillary voltage 3500 V, nebulizer 0.8 bar N_2_, dry temperature 200 °C, and dry gas flow 7.0 L/min N_2_. The full-scan mass spectra were recorded in the range of *m*/*z* 50–550. To ensure the accuracy of mass spectrometer, calibration of the instrument with a calibration solution for small molecules (Agilent Technologies, Santa Clara, CA, USA) was performed before each measurement. The concentration of the test compounds in the rat plasma and CSF were calculated using calibration curves in the rat plasma and rat tissue samples (brain, liver, kidney, and spinal cord) with 1:9 rat whole brain homogenate in PBS (*w*:*v*) (see the Section 4.11).

### 4.11. Validation of Analytical Method

In-house partial validation of the analytical method was performed according to ICH Harmonised Tripartite Guidelines Q2(R1) and M10 [51,52]. The following validation parameters were determined: selectivity, specificity, linearity, accuracy, precision, the limit of quantification (LOQ), and the limit of detection (LOD). All validation experiments were carried out for all matrices by using a drug-free pooled matrix to ensure accurate results. Selectivity was manually assessed by examining non-spiked pooled matrix samples for potentially interfering peaks. Specificity was evaluated by spiking blank matrix samples with *R*-modafinil as quality control (QC) samples and its metabolite modafinic acid (1000 ng/mL) and measuring the accuracy of *R*-modafinil, which was set to be within ±15% of the nominal values. The linearity of the calibration curve for each analyte was plotted as the ratio of the analyte (A) and IS areas (Area A/IS) on the x axis and as the ratio of A and IS concentration (C A/IS) on y axis. It was assessed using the regression coefficient (R^2^) and using data from three sets of independently prepared calibration standards, which were prepared and analyzed on three different days in triplicate each. The concentration of the IS was 500 ng/mL, or, in the case of HLM incubation, 1 µM. The working range for both analytes was derived from linearity and established by achieving an acceptable level of linearity, precision, and accuracy in the range of 20–2000 ng/mL in each matrix. The accuracy, repeatability, and intermediate precision were estimated in triplicates for each analyte at initial concentrations within the validation range by using QC samples at three levels of low, medium, and high (50, 500, and 1000 ng/mL). The accuracy was calculated by comparing the means of the measured analyte concentrations of the QC samples to their theoretical content and was expressed as relative error (RE%). Repeatability, also addressed as intra-day variation of an assay, was calculated by measuring the prepared QC samples twice within the same batch. The intermediate precision or inter-day variation of an assay was calculated by remeasuring the same set of samples on three different days. The repeatability and intermediate precision were expressed as relative standard deviation (RSD%). The signal suppression or enhancement caused by the matrix effects (ME) and SPE recovery (RE) was calculated for all matrices from peak areas of the spiked samples, post-preparation spiked samples, and non-extracted neat solvent samples using the QC samples. For each analyte in a specific matrix, the LOD and LOQ values were determined based on signal-to-noise (S/N) ratios of 3 and 10, respectively.

In vitro plasma stability assay was used in the scope of the validation studies. Briefly, 40 μL of blank rat plasma or blank rat plasma containing 10% DMF as an inhibitor was first pre-warmed for 5 min at 37 °C using a Thermomixer^®^ comfort (Eppendorf, Hamburg, Germany). The assay was initiated by the addition of 10 μL of *R*-modafinil (1000 ng/mL). The reaction was terminated after 0, 1, 2, and 4 h, via the addition of 50 μL of ice-cold ACN containing the IS solution. The samples were centrifuged for 10 min at 3000× *g* at 4 °C. The supernatant of all samples was diluted with 0.1% FA in water, 1:2 (*v*:*v*), and 10 µL of the sample was injected onto the LC–HRMS system. The plasma stability of *R*-modafinil was expressed by calculating the percentage (%) of the parent drug remaining after incubation and plotted against the incubation time.

### 4.12. Data Analysis

Interpretation of the generated data, statistical analysis, and graph/figure generation were performed using Microsoft Excel 2016 (Microsoft Corporation, Redmond, WA, USA) and GraphPad Prism version 8.0.1 for Windows (GraphPad software, San Diego, CA, USA. The acquired LC–HRMS data were processed using Compass DataAnalysis (Bruker Daltonics, version 4.2). An untargeted workflow for the detection of metabolites was used with the metabolomics software MZmine 3.4.27 [81]. The raw data generated during the LC–HRMS measurements (data format .d) were converted into an open format (data format .mzML) using MSConvert Version 3.0 (ProteoWizard) [82]. The workflow included mass detection → feature processing (chromatogram building/ADAP chromatogram builder) → feature alignment → gap-filing → export [83,84,85,86]. The settings for each step are described in Supplementary Materials in Table S5.

## 5. Conclusions

In summary, by combining relevant in vivo and in vitro methods with a robust LC–HRMS method, we have accurately analyzed the distribution of *S*-CE-123 and *R*-modafinil within the CNS in rats and their metabolism in HLMs. Our data highlight the differences in the unbound concentrations in both rat plasma and brain ISF, as well as their hepatic metabolism in HLMs and rat plasma stability. Importantly, for the first time, we demonstrate that the novel DAT inhibitor *S*-CE-123 crosses the BBB to a greater extent than *R*-modafinil does, as indicated by its higher K_p,uu,brain_ values. Furthermore, *S*-CE-123 is found to predominantly reside in the brain interstitial space, while *R*-modafinil appears to distribute more evenly across both sides of the plasma membrane of the brain parenchymal cells (K_p,uu,cell_). This suggests an increased presence of *S*-CE-123 at the target brain interstitial site. Moreover, our study has led to some unexpected findings, including the detection of metabolites of both compounds in brain and spinal cord samples. Additionally, we showed *R*-modafinil’s instability in rat plasma, which must be considered when designing PD studies utilizing modafinil as a reference drug. These findings suggest that further research is required to understand the roles of the metabolites and to clarify the metabolism of *R*-modafinil. In conclusion, this study provides an essential foundation for further PKPD research on the novel modafinil analog *S*-CE-123.

## Figures and Tables

**Table 1:** Method validation parameters for *R*-modafinil and *S*-CE-123 including concentration range, coefficient of determination of linear regression model (R^2^), accuracy (RE%), repeatability (RSDr), intermediate precision (RSDip), limit of detection (LOD), limit of quantification (LOQ), recovery (RE), and matrix effect (ME) (n = 3, mean).

Analytes	Matrix	Range	R^2^	Level	RE%	RSDr	RSDip	LOD	LOQ	RE	ME
		ng/mL		ng/mL	%	%	%	ng/mL	ng/mL	%	%
*R*-MO	Plasma	20–2000	0.999	50	−2.1	10.2	12.9	10	20	112 ± 18	113 ± 40
				500	1.2	7.5	11.4			94 ± 11	73 ± 4
				1000	1.4	5.7	5.5			95 ± 10	74 ± 7
	Brain *	20–2000	0.998	50	2.0	7.0	2.9			98 ± 28	107 ± 5
				500	−6.2	8.6	12.5			96 ± 5	93 ± 11
				1000	6.7	3.4	8.2			109 ± 6	71 ± 7
*S*-CE-123	Plasma	20–2000	0.995	50	−0.3	3.9	8.9	10	20	102 ± 16	103 ± 16
				500	−6.6	7.6	11.1			97 ± 7	74 ± 7
				1000	−1.4	6.3	9.5			91 ± 14	78 ± 11
	Brain *	20–2000	0.999	50	14.2	5.5	11.3			99 ± 12	97 ± 13
				500	−9.1	2.6	3.8			98 ± 15	88 ± 24
				1000	−0.5	2.4	3.5			102 ± 2	70 ± 5

* Brain matrix refers to 1:9 rat whole brain homogenate in phosphate-buffered saline (PBS), pH 7.4 (*w*:*v*); *R*-MO—*R*-modafinil; RE%—accuracy; RSDr—repeatability; RSDip—intermediate precision; RE—recovery; ME—matrix effect.

**Table 2:** Summary of brain distribution and systemic PK parameters of *S*-CE-123 and *R*-modafinil.

Parameter	Unit	*S*-CE-123	*R*-modafinil
Total brain-to-plasma concentration ratio, K_p,brain_	unitless	0.61 ± 0.10	0.29 ± 0.013
Unbound brain-to-plasma concentration ratio, K_p,uu,brain_	unitless	0.46 ± 0.032 ^§^	0.097 ± 0.034 ^§^
Total steady-state plasma concentration, C_plasma,ss_	nM	4149 ± 2202	5574 ± 649
Fraction unbound in plasma, f_u,plasma_	unitless	0.252 ± 0.023	0.797 ± 0.15
Unbound volume of distribution in brain, V_u,brain_	mL/g brain	5.21 ± 1.12	3.73 ± 1.38
Fraction unbound in brain homogenate, f_u,brain_	unitless	0.035 ± 0.012	0.23 ± 0.10
Unbound intracellular-to-extracellular (interstitial) concentration ratio, K_p,uu,cell_	unitless	0.18 ± 0.09 ^§^	0.85 ± 0.6 ^§^

^§^—standard deviation was calculated on the basis of the error propagation method (see Section 4.7).

## Data Availability

The data presented in this study are available within this article or Supplementary Materials.

## Supplementary Materials

The supporting information can be downloaded at https://www.mdpi.com/article/10.3390/ijms242316956/s1.
