# Supplementary material for: Pharmacokinetics of Novel Dopamine Transporter Inhibitor CE-123 and Modafinil with a Focus on Central Nervous System Distribution
Source: Int J Mol Sci. 2023 Nov 29;24(23):16956. doi: 10.3390/ijms242316956 (PMC10707468; doi:10.3390/ijms242316956)
Supplement: Supplementary file 1 [file ijms-24-16956-s001.zip › ijms-2731679-supplementary.pdf]

Supplementary Material

Pharmacokinetics of Novel Dopamine Transporter Inhibitor, CE-123 and Modafinil with a Focus on Central Nervous System Distribution

Iva Spreitzer [1,2], Josefin Keife [3], Tobias Strasser [1], Predrag Kalaba [1], Jana Lubec [4], Winfried Neuhaus [5,6], Gert Lubec [4], Thierry Langer [1], Judith Wackerlig [1,*] and Irena Loryan [3,*]

[1] Department of Pharmaceutical Sciences, University of Vienna, 1090 Vienna, Austria
[2] Vienna Doctoral School of Pharmaceutical, Nutritional and Sport Sciences, University of Vienna, 1090, Vienna, Austria.
[3] Translational Pharmacokinetics/Pharmacodynamics Group, Department of Pharmacy, Uppsala University, Uppsala, 75123, Sweden
[4] Programme for Proteomics, Paracelsus Medical University, 5020 Salzburg, Austria
[5] Competence Unit Molecular Diagnostics, Center Health and Bioresources, AIT Austrian Institute of Technology GmbH, Vienna, Austria
[6] Department of Medicine, Faculty of Medicine and Dentistry, Danube Private University, 3500 Krems, Austria

* Correspondence: JW judith.wackerlig@univie.ac.at; IL irena.loryan@farmaci.uu.se

Table of content

**Figure S1.** Plasma stability of *R*-modafinil after 4-h incubation in rat plasma and rat plasma containing 10 % DMF as an inhibitor (n=3, mean ± SD).	3
**Table S1.** Assessment *R*-modafinil's (50, 500 and 1000 ng/mL) accuracy in presence of modafinic acid (1000 ng/mL) (n=3, mean ± SD).	3
**Figure S2. A:** Extracted ion chromatogram of *S*-CE-123 metabolite M1 at *m/z* 330.0624 after 60 minutes of incubation of *S*-CE-123 (50 μM) with human liver microsomes. **B:** High resolution mass specter of *S*-CE-123 metabolite M1.	4
**Table S2.** Total concentration of *S*-CE-123 and *R*-modafinil in rat plasma, brain, CSF, liver, kidney, and spinal cord 4 hours intravenous constant infusion of 20 mg/kg each (n=3, except CSF n=2 for *S*-CE-123 and n=1 for *R*-modafinil).	5
**Figure S3.** Relative exposure of M1, metabolite of *S*-CE-123 and modafinil acid (MA) and modafinil sulfone (MS), metabolites of *R*-modafinil in rat plasma and CSF and vital organs after 4-hour intravenous constant infusion of 20 mg/kg of *S*-CE-123 and *R*-modafinil (n=3, except CSF n=2 for *S*-CE-123 and n=1 for *R*-modafinil, mean ± SD)	6
**Figure S4.** Extracted ion chromatograms of *R*-modafinil (peak 1) and modafinil sulfone (peak 2) at *m/z* 167.0841 (red) and IS (peak 3) at *m/z* 328.0864 (green) in rat brain after 4-hour intravenous constant infusion of R-modafinil (20 mg/kg) compared to total ion chromatogram of a blank rat brain (blue). The concentration of R-modafinil in sample is 471 ng/mL and IS 500 ng/mL.	7
**Table S3.** Estimated unbound plasma concentrations ($C_{u,plasma}$) and unbound-drug concentration in brain interstitial fluid ($C_{u,brain,ISF}$) during 4-h intravenous constant infusion of 20 mg/kg of *S*-CE-123 and *R*-modafinil.	8
**Figure S5.** Structural model used for simulation of time concentration profiles of *S*-CE-123 and *R*-modafinil.	9
**Figure S6.** Simulated unbound plasma, unbound brain and total plasma concentration-time profiles of *S*-CE-123 and *R*-modafinil obtained during 4-h intravenous administration of *S*-CE-123 and *R*-modafinil and additional 3 hours post-infusion.	9
**Figure S7.** Relative exposure-time profile reflecting formation of M1, metabolite of *S*-CE-123 as well as modafinil acid (MA) and modafinil sulfone (MS), metabolites of *R*-modafinil in plasma during 4-hour intravenous constant infusion of 20 mg/kg of *S*-CE-123 and *R*-modafinil (n=3 rats, per compound, mean ± SD).	10
**Table S4.** Parameters used in simulation exercise.	11
**Table S5.** MZmine 3 processing parameters.	12

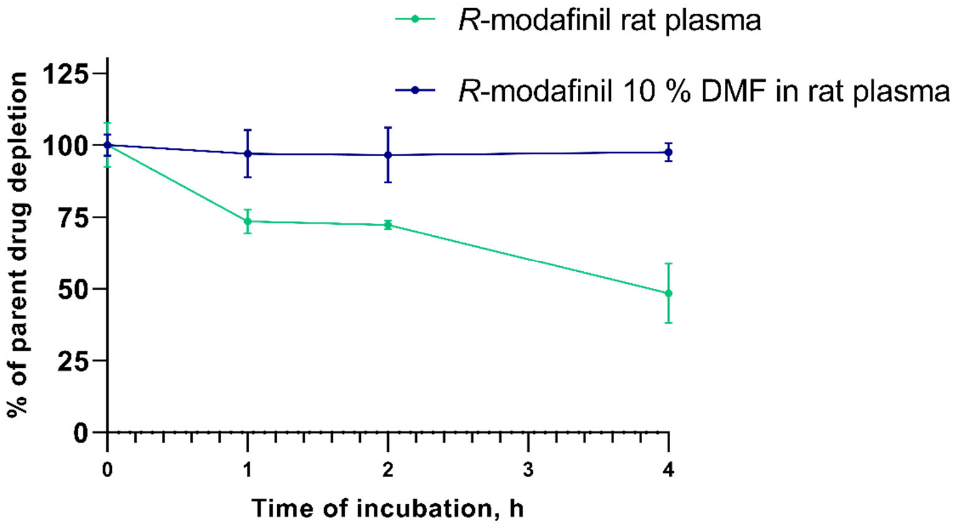

Figure S1. Plasma stability of *R*-modafinil after 4-h incubation in rat plasma and rat plasma containing 10 % DMF as an inhibitor (n=3, mean ± SD).

Table S1. Assessment *R*-modafinil's (50, 500 and 1000 ng/mL) accuracy in presence of modafinic acid (1000 ng/mL) (n=3, mean ± SD).

Level ng/mL	Accuracy RE% [%]
1000	7.7 ± 8.7
500	-3.4 ± 7.6
50	9.8 ± 9.8

A

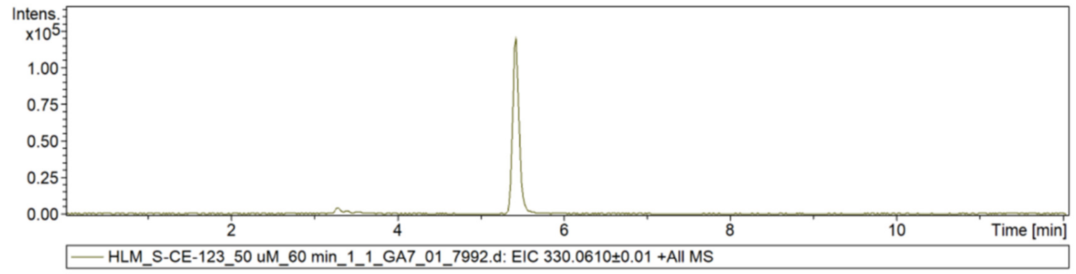

B

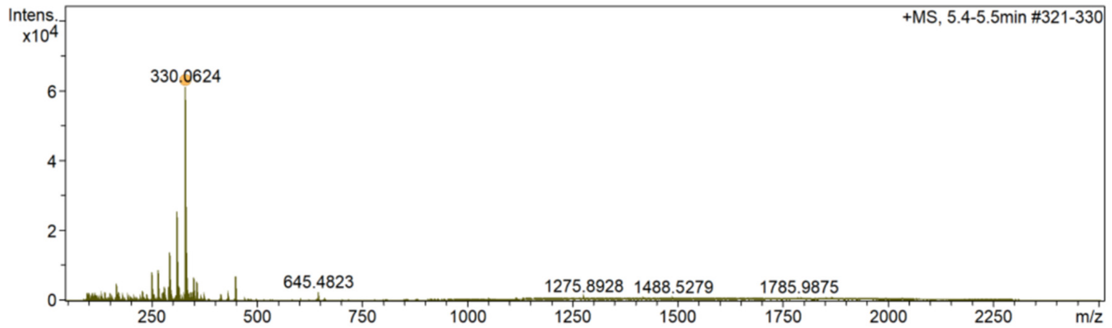

Meas. m/z	#	Ion Formula	m/z	err [ppm]	mSigma	# mSigma	Score	rdb	e⁻ Conf	N-Rule
330.0624	1	C17H16NO2S2	330.0617	-2.0	14.5	1	100.00	10.5	even	ok

Figure S2. A: Extracted ion chromatogram of *S*-CE-123 metabolite M1 at *m*/*z* 330.0624 after 60 minutes of incubation of *S*-CE-123 (50 μM) with human liver microsomes. **B:** High resolution mass specter of *S*-CE-123 metabolite M1.

Table S2. Total concentration of *S*-CE-123 and *R*-modafinil in rat plasma, brain, CSF, liver, kidney, and spinal cord 4 hours intravenous constant infusion of 20 mg/kg each (n=3, except CSF n=2 for *S*-CE-123 and n=1 for *R*-modafinil).

	***S*-CE-123 (n=3)**	***R*-modafinil (n=3)**
**Plasma**		
Conc., ng/mL	1052	1008
SD ng/mL	561	210
CV %	53	21
**CSF**		
Conc., ng/mL	570* and 104**	784
SD ng/mL	-	-
CV %	-	-
**Brain**		
Conc., ng/g	820	448
SD ng/g	483	31
CV %	59	7
**Spinal cord**		
Conc., ng/g	1244	733
SD ng/g	685	72
CV %	55	10
**Liver**		
Conc., ng/g	2727	2170
SD ng/g	1621	724
CV %	59	33
**Kidney**		
Conc., ng/g	1785	2536
SD ng/g	1047	466
CV %	59	18

*Rat 1; **Rat 2

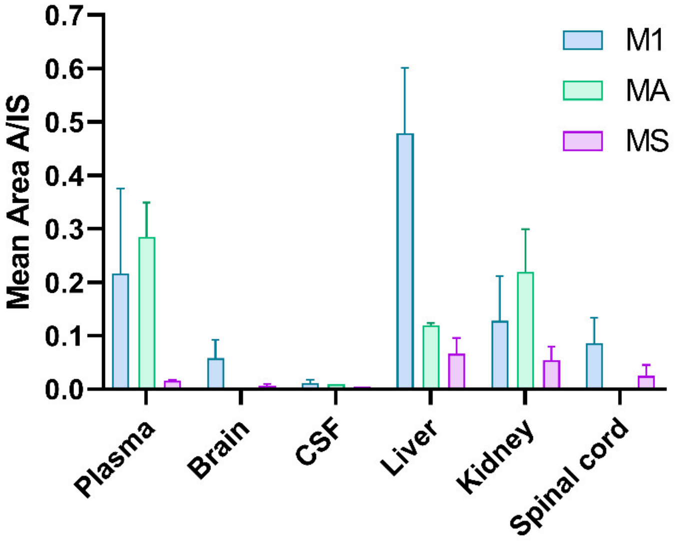

Figure S3. Relative exposure of M1, metabolite of *S*-CE-123 and modafinil acid (MA) and modafinil sulfone (MS), metabolites of *R*-modafinil in rat plasma and CSF and vital organs after 4-hour intravenous constant infusion of 20 mg/kg of *S*-CE-123 and *R*-modafinil (n=3, except CSF n=2 for *S*-CE-123 and n=1 for *R*-modafinil, mean ± SD)

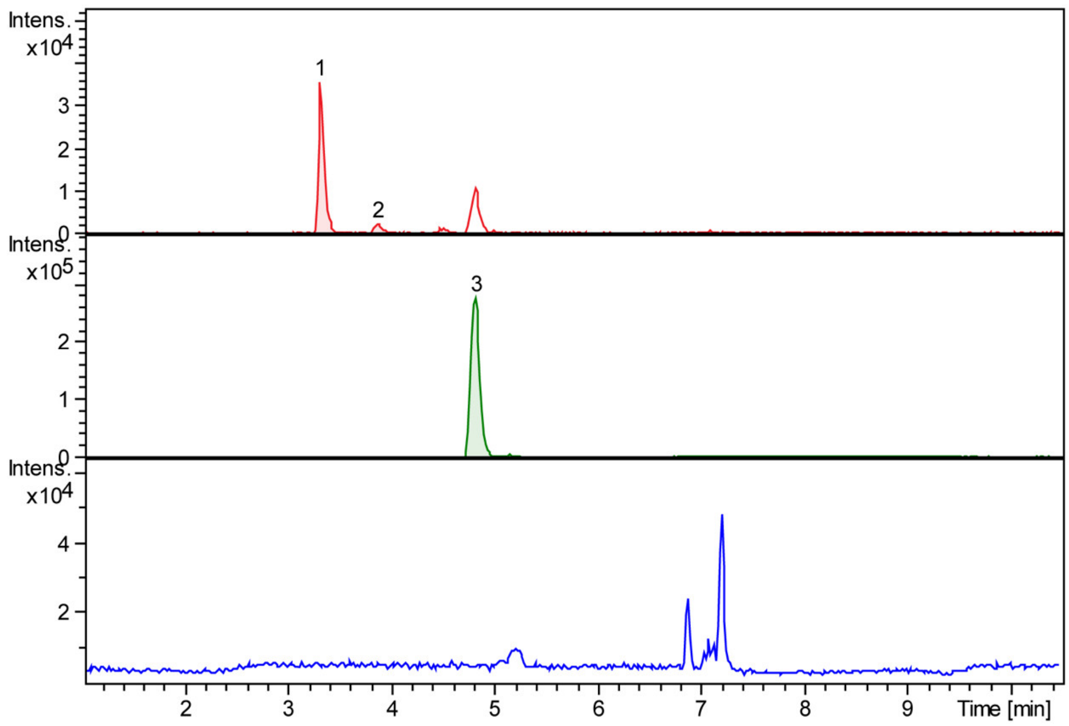

Figure S4. Extracted ion chromatograms of *R*-modafinil (peak 1) and modafinil sulfone (peak 2) at *m/z* 167.0841 (red) and IS (peak 3) at *m/z* 328.0864 (green) in rat brain after 4-hour intravenous constant infusion of *R*-modafinil (20 mg/kg) compared to total ion chromatogram of a blank rat brain (blue). The concentration of *R*-modafinil in sample is 471 ng/mL and IS 500 ng/mL.

Table S3. Estimated unbound plasma concentrations ($C_{u,plasma}$) and unbound-drug concentration in brain interstitial fluid ($C_{u,brain,ISF}$) during 4-h intravenous constant infusion of 20 mg/kg of *S*-CE-123 and *R*-modafinil.

	Parameters	Value	Unit
S-CE-123	Fraction unbound in plasma, $f_{u,plasma}$	0.25	unitless
	Unbound brain-to-plasma concentration ratio, $K_{p,uu,brain}$	0.46	unitless
	Total drug concentration in plasma, $C_{tot,plasma}$	3355	nM
	Unbound-drug concentration in plasma, $C_{u,plasma}$	839	nM
	Unbound-drug concentration in brain interstitial fluid, $C_{u,brainISF}$	386	nM
	Half-maximal inhibitory concentration, IC_{50} [a]	4600	nM
	Inhibitory constant, K_i [b]	610	nM
R-modafinil	Fraction unbound in plasma, $f_{u,plasma}$	0.79	unitless
	Unbound brain-to-plasma concentration ratio, $K_{p,uu,brain}$	0.097	unitless
	Total drug concentration in plasma, $C_{tot,plasma}$	3686	nM
	Unbound-drug concentration in plasma, $C_{u,plasma}$	2912	nM
	Unbound-drug concentration in brain interstitial fluid, $C_{u,brainISF}$	282	nM
	Half-maximal inhibitory concentration, IC_{50} [c]	4000	nM
	Inhibitory constant, K_i [c]	780	nM

a – Data from Kristofova *et al.* 2018 [36];
b – Data from Lubec *et al.* 2023 [56];
c – Data from Loland *et al.* 2012 [27];

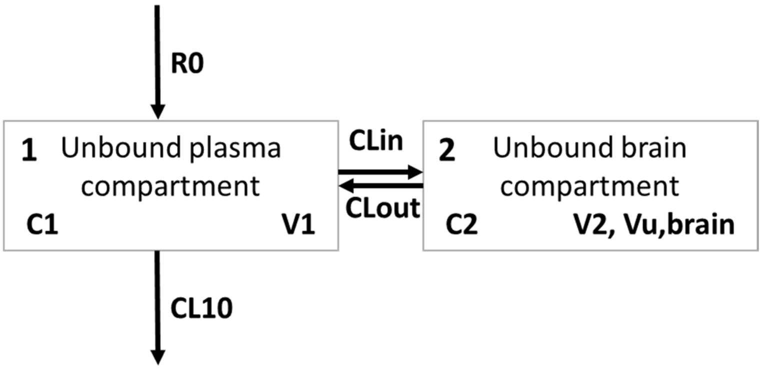

Figure S5. Structural model used for simulation of time concentration profiles of *S*-CE-123 and *R*-modafinil.

Figure S6. Simulated unbound plasma, unbound brain and total plasma concentration-time profiles of *S*-CE-123 and *R*-modafinil obtained during 4-h intravenous administration of *S*-CE-123 and *R*-modafinil and additional 3 hours post-infusion.

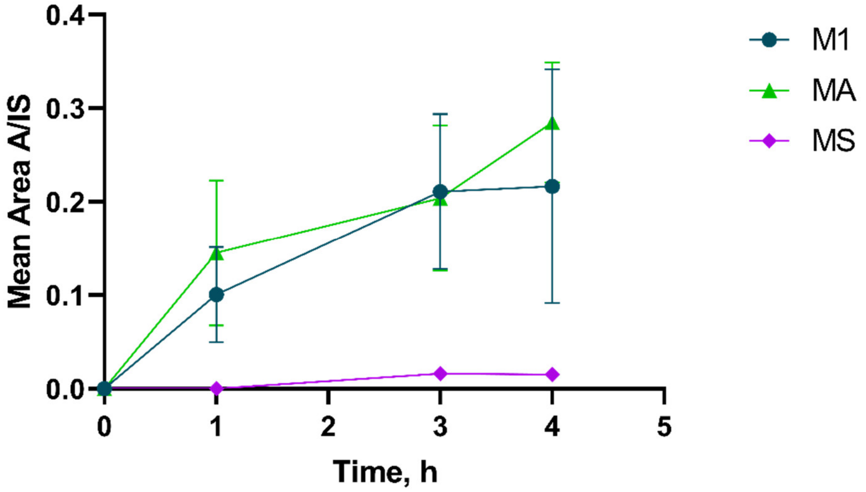

Figure S7. Relative exposure-time profile reflecting formation of M1, metabolite of *S*-CE-123 as well as modafinil acid (MA) and modafinil sulfone (MS), metabolites of *R*-modafinil in plasma during 4-hour intravenous constant infusion of 20 mg/kg of *S*-CE-123 and *R*-modafinil (n=3 rats, per compound, mean ± SD)

Table S4. Parameters used in simulation exercise.

Parameter	Unit	*S*-CE-123	*R*-modafinil
**Systemic parameters**			
**Systemic clearance, *CL***	L/h	0.184	1.498
**Systemic clearance unbound, *Clu***	L/h	0.046	1.194
**Apparent volume of distribution, V_d**	L	0.232	1.022
**Apparent volume of distribution unbound, V_d**	L	0.058	0.814
**Fraction of unbound in plasma, $f_{u,plasma}$**	unitless	0.252	0.797
**Rate of BBB transport parameters***			
**Pe**	cm/min	0.0030	0.0023
**Pe rat****	mL/min	0.545	0.422
**Clin**	L/h	0.033	0.025
**Clout**	L/h	0.071	0.261
**Extent of BBB transport and intra-brain distribution**			
**Unbound brain-to-plasma concentration ratio, $K_{p,uu,brain}$**	unitless	0.46	0.097
**Unbound volume of distribution in brain, $V_{u,brain}$**	mL/g brain	5.21	3.73
**Unbound volume of distribution in brain, $V_{u,brain}$**	L	0.009378	0.006714
**Additional parameters used**			
**Infusion rate**	mg/h	0.05	1.0
**Rat weight**	kg	0.3	0.3
**Brain weight**	g	1.8	1.8
**Surface area of BBB**	cm^2	180	180
**MW**	Da	313.1	273.4

* Data of blood-brain barrier permeation of 100 μM CE-123 and modafinil across an *in vitro* Transwell model based on mouse cell line cerebEND were included in the simulation. Experimental procedures were published previously [36,40] The permeability coefficients were calculated according to the clearance principle deducting blank inserts without cells.

**Similar apparent permeability in human BBB cell culture model and rat endothelial cells was assumed.

Table S5. MZmine 3 processing parameters.

Parameter	Mass detection	Chromatogram building	^{13}C isotope filter	Join aligner
Mass detection	Centroid			
Noise level	1.00E+03			
MS level	1			
Min consecutive scans		5		
Min intensity for consecutive scans		1.00E+04		
m/z tolerance		0.001 (5 ppm)	0.001 (5 ppm)	0.001 (5 ppm)
Chromatographic threshold (%)				
Search minimum in RT[a] range (min)				
Minimum relative height (%)				
Min ratio of peak top/edge				
Peak duration range (min)				
RT tolerance (absolute, min)			0.01	
Maximum charge			1	
Representative isotope			lowest *m/z*	
Weight for *m/z*				10
RT tolerance (relative, %)				5
Weight for RT				5

a – RT refers to retention time
